# Supplementary figures and images for: Physical Blending of Fractionated Bambangan Kernel Fat Stearin and Palm Oil Mid-Fraction to Formulate Cocoa Butter Equivalent
Source: Foods. 2023 Apr 23;12(9):1744. doi: 10.3390/foods12091744 (PMC10178490; doi:10.3390/foods12091744)

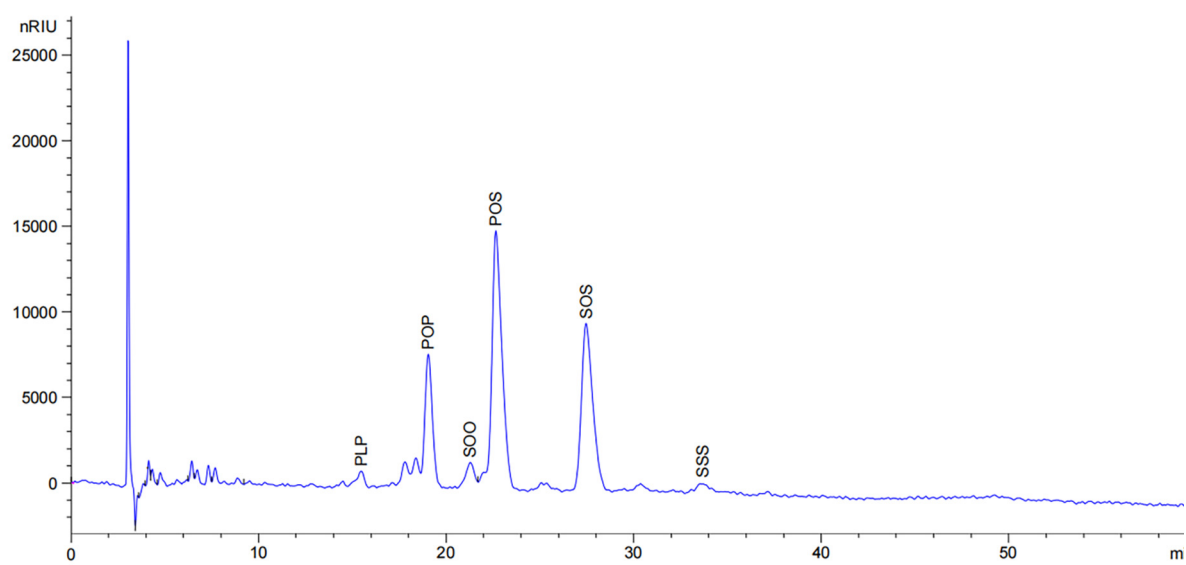

**CB**

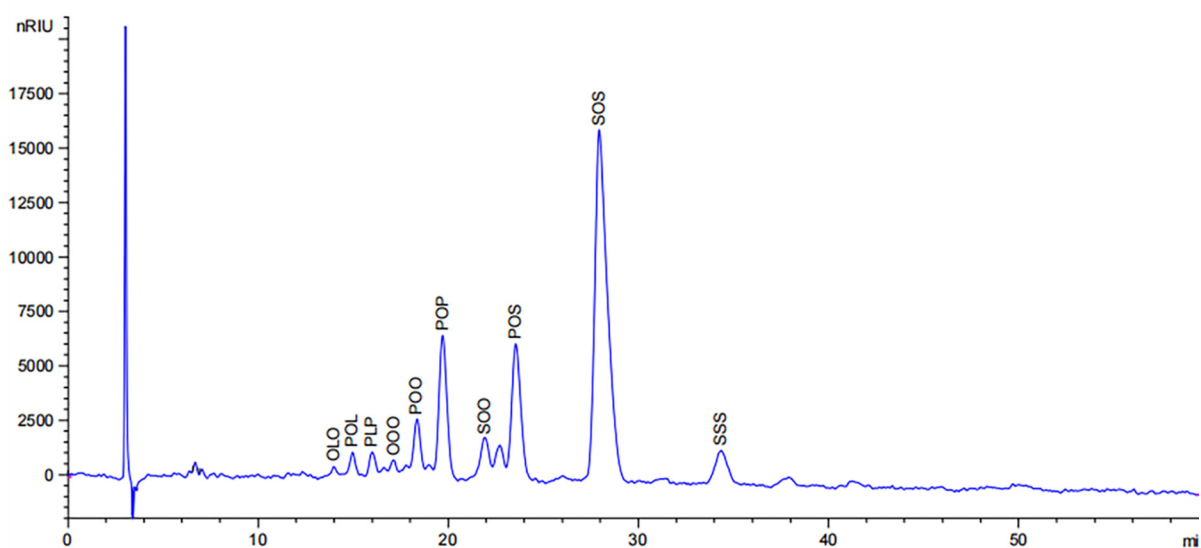

**B5**

**Figure S1.** Representative HPLC chromatogram of TAG in CB and B5.

Supplement: Supplementary file 1 [file foods-12-01744-s001.zip › foods-2176531-supplementary.pdf]
